# Heineke-Mikulicz Principle in a Male With Failed Recurrent Urethral Diverticulum

**DOI:** 10.7759/cureus.67972

**Published:** 2024-08-27

**Authors:** Saravanan Jambunathan, Balaji Subramaniam

**Affiliations:** 1 Urology, Sri Ramaswamy Memorial (SRM) Medical College Hospital and Research Centre, Chennai, IND

**Keywords:** heineke-mikulicz, urethral calculus, recurrent diverticulum, urethroplasty, urethral diverticulum, calculus

## Abstract

Urethral diverticulum with a calculus in a male patient is an uncommon phenomenon, in which the management plan differs from one case to another. The formation of calculi can be attributed to the long-standing urinary stasis in the diverticulum leading to recurrent urinary tract infection and stone formation. In our case, a 35-year-old male was treated multiple times with excision and repair of urethral diverticulum leading to recurrent diverticula and calculus formation. He was successfully managed with excision of the defect followed by reconstruction using the Heineke-Mikulicz principle. No recurrence was noted during long-term follow-up.

## Introduction

Urethral calculus along with a diverticulum in a male patient is a rare entity for which the management is considered tricky. Although many cases have been reported in the literature regarding urethral diverticula with calculi in females, the number of cases in the male population is subsequently less [1,2]. The consequences of urethral diverticulum in a male are often related to inadequate urethral diverticulum drainage, with the urethral diverticulum acting as a nidus for urinary stasis. This, in turn, leads to recurrent urinary tract infection, stone formation, a subsequent increase in the urethral diverticulum size, urinary leakage, incontinence, or a palpable peno-scrotal mass [3]. Acquired urethral diverticula often result from stricture, infection, or trauma. We present our experience with an acquired recurrent failed urethral diverticulum in a male, who was managed using a longitudinal incision followed by a transverse closure (Heineke-Mikulicz principle). 

## Case presentation

**Figure 1:**
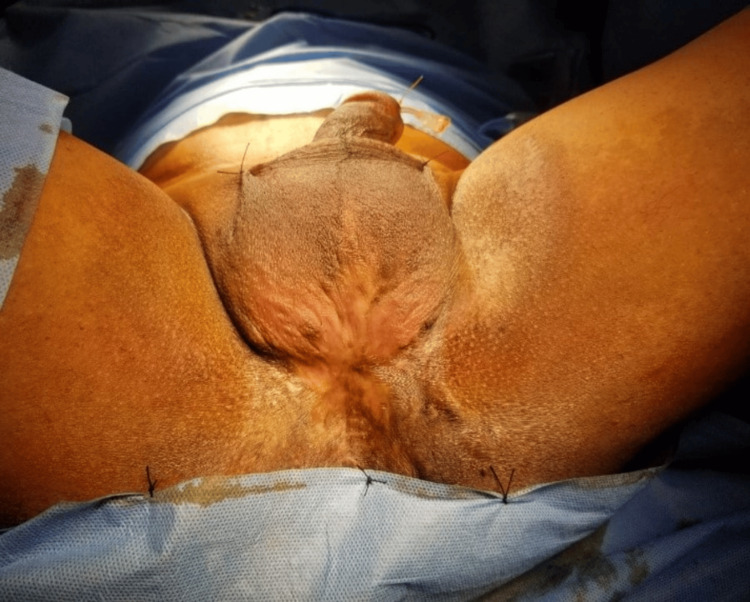
Severely scarred perineal region due to multiple previous interventions

**Figure 2:**
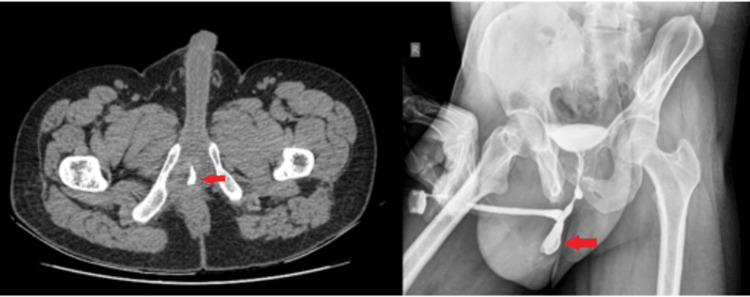
(Left) Computed tomography image suggestive of diverticulum with calculus. (Right) AUG image suggestive of diverticulum containing calculus AUG: ascending urethrogram

A 35-year-old male presented with a history of pelvic injury leading to urinary retention, for which he was treated with an initial suprapubic urinary diversion followed by perineal urethroplasty. Subsequently, a staged urethroplasty was done with the help of a tubularised scrotal skin flap. Later on, he was evaluated for poor urinary stream and diagnosed to have a urethral diverticulum in the junction between the flap and urethral mucosa on ascending urethrogram (AUG). He was managed twice with excision of the diverticulum in the past six years. Currently, he presented with lower urinary tract symptoms (LUTS) and recurrent urinary tract infection along with a severely scarred perineal region, as shown in Figure 1. On evaluation with AUG and computed tomography (CT), he was diagnosed with recurrent urethral diverticulum, which was filled with calculus as shown in Figure 2. Cystoscopy was done, and encrusted hair in the diverticular lumen was noted.

**Figure 3:**
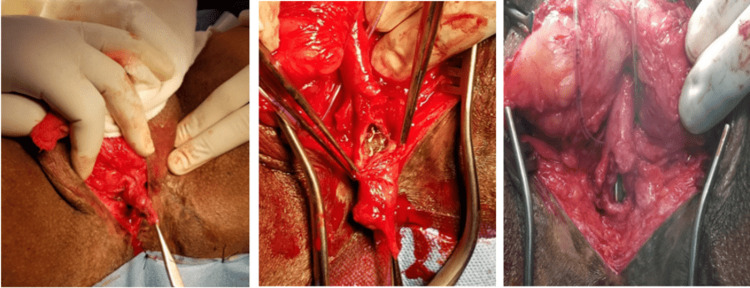
(A) Diverticular sac dissected. (B) Sac opened showing multiple calculi with an encrusted tuft of hair. (C) The diverticular sac excised and repaired using the Heineke-Mikulicz principle

The patient was then taken up for diverticular excision and clearance of all hair and calculus, which left a longitudinal defect, for which closure was done in a transverse fashion, based on the Heineke-Mikulicz principle as shown in Figure 3.

**Figure 4:**
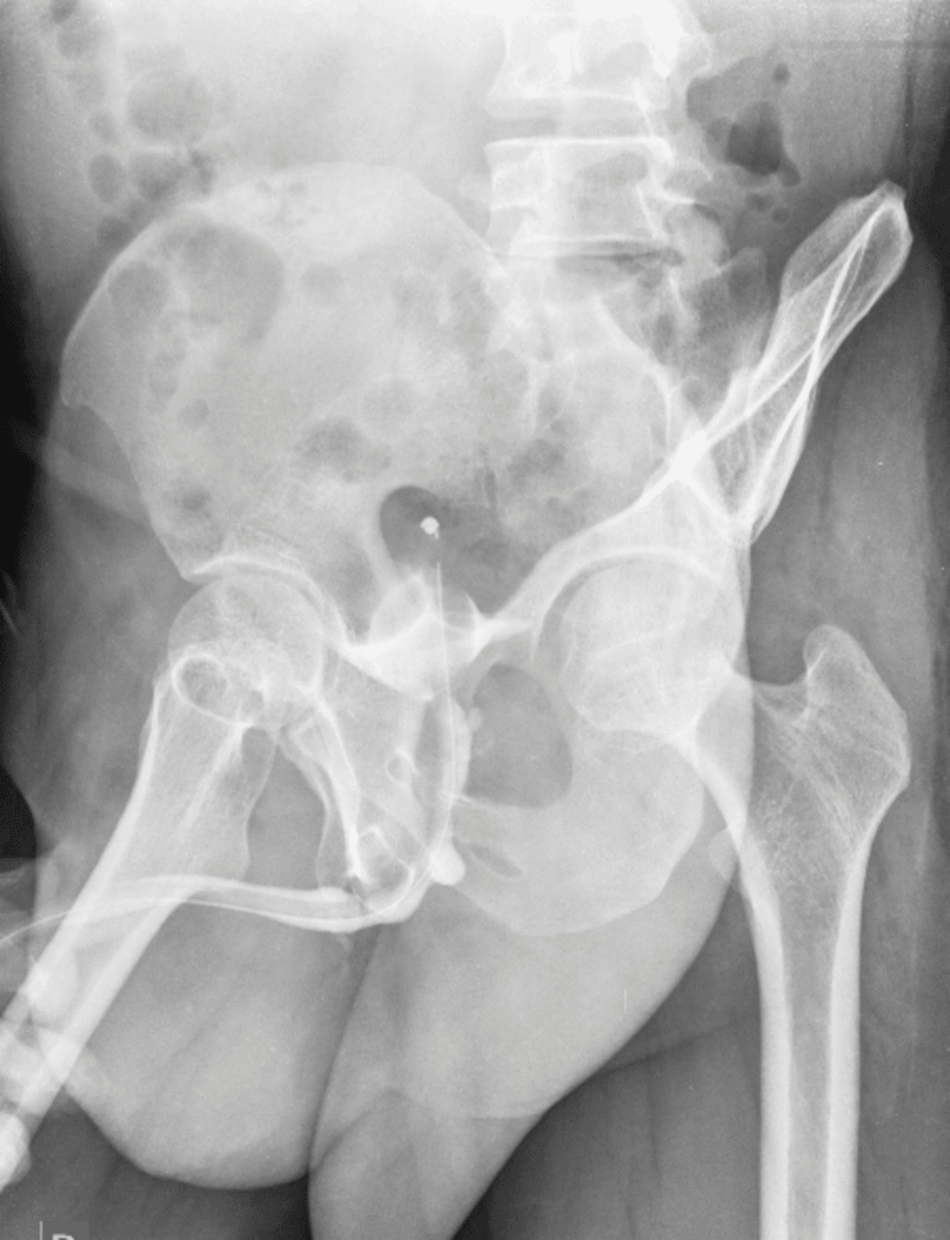
AUG image showing normal post-operative urethra AUG: ascending urethrogram

Post-operative recovery was uneventful. After catheter removal, the patient voided freely. Post-operatively, AUG showed normal filling (Figure 4), and uroflowmetry showed a maximum voided rate of 14 ml/sec. Follow-up check cystoscopy after three months showed a well-healed anastomosis site and adequate urethral lumen.

## Discussion

The common areas of urethral calculi are noted in the fossa navicularis, bulbar, and prostatic urethra [4]. While few reports have mentioned about the high occurrence of calculi in the anterior urethra, there are a large number of cases of posterior urethral calculi as well [5]. Usually, these urethral calculi are of secondary origin, which commonly descends from the upper urinary tract. However, in the case of associated urethral stricture, enlarged prostate, or urethral diverticulum, there is an associated risk of urethral calculus [6]. A urethral diverticulum has been known to be treated by various surgical modalities, such as diverticulum excision and reconstruction and urinary diversion methods, or by non-operative methods [7]. The plan of management for urethral calculus varies with the site, size, and shape of the calculus. Small calculi are usually expected to pass out spontaneously or can be extracted after instilling xylocaine jelly via the urethra [8]. The troublesome calculi are the ones which get lodged in the urethra causing urinary obstruction. Calculi near the meatus or fossa navicularis can be extracted out using meatotomy. The calculi noted in the posterior urethra can be pushed back into the bladder and can be retrieved by either open or endoscopic procedures. Few cases have been reported in literature where a calculus in the membranous urethra was removed completely followed by end-to-end urethroplasty, leading to successful results [9,10]. A similar case of large calculus in an anterior urethra diverticulum was treated with pneumatic combined with ultrasound lithotripsy [11]. In our case, the urethral diverticulum is considered to be the reason for the formation of the calculus, due to long-standing stasis causing recurrent diverticulum formation and infection. The urethral diverticulum containing the calculus was completely excised and repaired using the principle of Heineke-Mikulicz urethroplasty to avoid the recurrence of the diverticulum. The same principle has been attempted for multiple cases in urethroplasty and has been shown to have promising results [12].

## Conclusions

Urethral diverticula with remnant calculi can be commonly managed using urethroplasty. Recurrent diverticulum formation is a known complication after urethroplasty as well. Since there are minimal literature and limited treatment modalities in the management of failed urethroplasty, the treatment varies from case to case on an individualized basis. While the Heineke-Mikulicz principle is commonly used in the gastrointestinal reconstruction, its usage in a urological surgery is uncommon. Urethroplasty using the Heineke-Mikulicz principle has been shown to provide a good result in the case of recurrent failed excision of the urethral diverticulum, which gives a potential solution to a complex question.
